# Expression VIII: Final Results of the Individual Perception and Level of Information of Patients with Borderline Tumors of the Ovary

**DOI:** 10.3390/curroncol33090516

**Published:** 2026-08-28

**Authors:** Sara Alavi-Demirci, Laura N. Beckmann, Hannah Woopen, Clemens Liebrich, Yasemine Virk, Pauline Wimberger, Karol Kubiak, Anette Ligl-Löhner, Hans-Martin Enzinger, Vera Czolk, Georg Kunz, Mandy Mangler, Tilmann Lantzsch, Alexander Mustea, Susanne Fechner, Aline Burdack, Jürgen Terhaag, Dorothea Fischer, Cornelia Müller, Jalid Sehouli

**Affiliations:** 1 Charité–University Medical Center Berlin, corporate member of Freie Universität Berlin and Humboldt-Universität zu Berlin, Department of Gynecology, European Competence Center for Ovarian Cancer, 13353 Berlin, Germany; dralavidemirci@gmail.com (S.A.-D.); lauranaomibeckmann@googlemail.com (L.N.B.);; 2 North-Eastern German Society of Gynecological Oncology (NOGGO e.V.), 13359 Berlin, Germany; y.virk@hotmail.com; 3 Department of Obstetrics and Gynecology, Klinikum Wolfsburg, 38440 Wolfsburg, Germany; clemens.liebrich@harzklinikum.com; 4 Department of Gynecology and Obstetrics, Medical Faculty, University Hospital Carl Gustav Carus, Technische Universität Dresden, 01307 Dresden, Germany; pauline.wimberger@ukdd.de; 5 Department of Gynecology and Obstetrics, St. Franziskus Hospital Münster, 48145 Münster, Germany; karol.kubiak@sfh-muenster.de; 6 Department of Gynecology and Obstetrics, Diakonissen-Stiftungs-Krankenhaus Speyer, 67346 Speyer, Germany; anette.ligl-loehner@diakonissen.de; 7 Department of Gynecology, Frauenklinik am Klinikum am Bruderwald, Sozialstiftung Bamberg, 96049 Bamberg, Germany; hans-martin.enzinger@sozialstiftung-bamberg.de; 8 Department of Gynecology and Obstetrics, Brüderklinikum Julia Lanz, 68163 Mannheim, Germany; v.czolk@diako-mannheim.de; 9 Department of Gynecology and Obstetrics, St. Johannes Hospital Dortmund, 44137 Dortmund, Germany; georg.kunz@joho-dortmund.de; 10 Department for Obstetrics and Gynecology, Auguste-Viktora-Krankenhaus Vivantes, 12157 Berlin, Germany; mandy.mangler@vivantes.de; 11 Department of Gynecology, Hospital St. Elisabeth and St. Barbara, 06110 Halle (Saale), Germany; t.lantzsch@krankenhaus-halle-saale.de; 12 Department of Gynaecology and Gynaecological Oncology, University Medical Center Bonn (UKB), 53127 Bonn, Germany; alexander.mustea@ukbonn.de; 13 German Ovarian Cancer Foundation, 13359 Berlin, Germany; susanne.fechner@avisomed.de; 14 Department of Gynecology and Obstetrics, Sana-Clinics Berlin Lichtenberg, 10365 Berlin, Germany; aline.burdack@sana.de; 15 Department of Gynecology and Obstetrics, Rottal Inn Kliniken, 84307 Eggenfelden, Germany; terhaag.juergen@rottalinnkliniken.de; 16 Department of Obstetrics and Gynaecology, Ernst von Bergmann Clinic, 14467 Potsdam, Germany; dorothea.fischer@klinikumevb.de; 17 Department of Gynaecology and Obstetrics, Brandenburg Medical School Theodor Fontane, University Clinic Brandenburg, 14770 Brandenburg an der Havel, Germany; c.mueller@klinikum-brandenburg.de

**Keywords:** borderline tumor, ovarian cancer, survey, doctor–patient relationship, information needs

## Abstract

**Background:** Borderline ovarian tumors (BOTs) represent a distinct group of rare epithelial ovarian neoplasms with excellent prognosis, but persistent uncertainty in patient understanding and clinical management. This study evaluated disease awareness, perceptions, and treatment patterns among affected women in Germany. **Methods:** The national multicenter Expression VIII survey was conducted across 34 centers by the North-Eastern German Society of Gynecologic Oncology (NOGGO). Using a standardized 46-item questionnaire, 286 patients with BOTs provided data on symptoms, treatment, fertility, follow-up, and illness perception. Descriptive analyses were performed. **Results:** Most participants (mean age 51 years) underwent surgery (94%), while systemic therapy was infrequent (chemotherapy 9%, bevacizumab 4%). Fertility-sparing surgery was performed in 14% overall, corresponding to 71% of those desiring future childbearing. Although 81% identified their physician as their main information source and rated information quality highly (mean 8.6/10), 29% believed they had ovarian cancer and 64% were unaware of their tumor stage. Nearly all patients (97%) received regular follow-up, though 18% were uncertain about the procedures performed. Disease severity was rated moderately high (mean 5.4/10), and recurrence risk and mortality were often overestimated. **Conclusions:** Despite good overall physician communication, substantial misconceptions persist among patients with BOTs, including the mistaken belief that they have been diagnosed with cancer, suggesting a need for clearer, structured education about its favorable prognosis and management of the disease. Improved guideline adherence and treatment centralization in specialized centers may reduce overtreatment and optimize fertility-preserving approaches.

## Simple Summary

Borderline ovarian tumors (BOTs) are rare epithelial ovarian neoplasms predominantly affecting younger women and generally associated with an excellent prognosis. Despite their favorable biology, many patients overestimate the malignant potential and recurrence risk of the disease. In this national multicenter survey of 286 women, most participants reported high satisfaction with physician-provided information, yet substantial knowledge gaps persisted regarding tumor type, stage, and prognosis. Variations in reported treatment and surgical approaches suggest ongoing uncertainty in clinical management. These findings underscore the importance of improved patient education, consistent implementation of guideline-based care, and management in specialized gynecologic oncology centers.

## 1. Introduction

Borderline ovarian tumors (BOTs) constitute a distinct group of rare epithelial ovarian neoplasms that histologically occupy an intermediate position between benign cystadenomas and malignant ovarian carcinomas. They are characterized by nuclear atypia in the absence of destructive stromal invasion [1,2,3]. BOTs account for approximately 10–20% of all epithelial ovarian tumors and are most commonly diagnosed at an early stage in the course of the disease [3,4,5]. Unlike invasive ovarian carcinoma, borderline ovarian tumors typically occur in a younger patient population, frequently in women of reproductive age, and are associated with a much more favorable prognosis, with five-year survival rates of 95% or higher across all stages combined [3,4,5,6,7]. The standard of care is surgical management with the objective of achieving macroscopic tumor clearance, while fertility-preserving procedures may be considered based on the patient’s age, menopausal status, and reproductive wishes [8,9,10,11,12]. The rarity of the disease, combined with its excellent overall prognosis, presents a considerable challenge for generating robust research data to define evidence-based therapeutic strategies. As a result, the current body of knowledge is derived predominantly from retrospective analyses of patient cohorts rather than from prospective or randomized studies [3,7,13]. This lack of robust, evidence-based data may contribute to a degree of uncertainty among treating physicians, which can in turn be reflected in patient care—most notably through insufficient patient counseling and a limited understanding of the disease and its therapeutic options [14,15,16,17,18]. The objective of the present study was to gain insight into the patient perspective and in particular to evaluate the extent of knowledge and awareness regarding their disease and therapeutic management among women diagnosed with this rare entity.

## 2. Materials and Methods

The Expression VIII study was a national multicenter survey conducted across 34 German centers using two standardized questionnaires. Both inpatients and outpatients receiving gynecologic or oncologic care were eligible for participation. The study was coordinated by the North-Eastern German Society of Gynecologic Oncology (NOGGO), which organized and distributed the paper-based questionnaires to participating centers. Patients’ subjective levels of information, perceptions, and physician–patient communication, among other topics, are assessed through anonymous self-report. As such, responses reflect patients’ personal understanding and recollection of their disease rather than objective clinical or pathological data, and were not cross-validated against electronic medical records. Two versions of the questionnaire were used: Form A for patients with low-grade ovarian carcinoma and Form B for patients with borderline ovarian tumors. Both versions contained 46 identical core questions, with an additional 4 items specific to low-grade patients in Form A and 3 items specific to borderline patients in Form B. Responses could be provided using multiple-choice options, percentage estimates, or ratings on a 10-point-rating scale, with 1 indicating “very poor” and 10 indicating “very good.” Depending on the question type, one or multiple responses were possible. Questionnaires were distributed at participating centers by the treating physician or study personnel, who confirmed eligibility based on a histopathological diagnosis of LGSC or BOT. No central registry was used to capture the total number of eligible patients for any participating center, and patients who declined to participate were not documented.

For the subgroup of patients with borderline ovarian tumors, data collection took place between March 2019 and November 2024. Eligible participants included all adult patients, irrespective of whether they were treated for primary diagnosis or recurrent disease. The study was approved by the respective national ethics committees (Charité Campus—Universitätsmedizin Berlin EA2/132/18). Written informed consent was not required, as participation was voluntary, anonymous, and completion of the questionnaire was considered implied consent. Completed questionnaires were returned to NOGGO by mail, where the data were entered into a secure web-based documentation system for centralized storage and analysis.

The present data represent the final analysis of the borderline ovarian tumor cohort. During phase I, a preliminary subanalysis of both the low-grade and borderline cohorts was conducted to define the key objectives for the final evaluation. The subanalysis and the final data of the low-grade cohort will be published independently in separate reports.

### Statistical Analysis

Data analysis was performed using Microsoft Excel (version 2024, Microsoft Corporation, Redmond, WA, USA). Results were analyzed descriptively and are presented as means ± standard deviation, medians with range, or modes, as appropriate. Categorical variables and frequency distributions are expressed as percentages to facilitate data interpretation. Missing responses did not lead to the exclusion of questionnaires. These were documented as “not answered,” and percentages for each item were calculated based on the total number of respondents (N) in each group. Ratings on the 10-point rating scale were treated as metric data and analyzed descriptively using measures of central tendency.

## 3. Results

### 3.1. Patient Characteristics

A total of 286 patients from 34 German cancer centers participated in the survey. The largest proportion were aged 18–41 years (26.6%). The age distribution showed a bimodal pattern, with peaks in the 18–41 (26.6%)- and 51–60 (25.2%)-year groups. The mean height was 166.1 ± 6.7 cm, and the mean weight was 75.2 ± 19.0 kg, corresponding to a mean BMI of 27.3 ± 6.7 kg/m^2^. At initial diagnosis, the majority underwent surgery (*n* = 266, 94.0%), while 17 (6%) did not (*n* = three missing responses). Reported procedures included oophorectomy (*n* = 229/266, 86.1%), hysterectomy (*n* = 109/266, 41%), omentectomy (*n* = 118/266, 44.4%), lymph node dissection (*n* = 38/266, 14.3%), bowel resection (*n* = 13/266, 4.9%), partial hepatectomy (*n* = 3/266, 1.1%), and other interventions (*n* = 66/266, 24.8%). Only a minority received adjuvant chemotherapy (*n* = 25, 9%), maintenance therapy with bevacizumab (*n* = 11, 4.2%), or antihormonal therapy (*n* = 6, 2.2%). In contrast, 252 (91%), 251 (95.8%), and 266 (97.8%) patients, respectively, did not receive these treatments, with small numbers of missing data. At the time of data collection, 210 patients (76.9%) had primary disease and 31 (11.4%) had recurrent disease, while 32 (11.7%) were unaware of their disease status and reported it as unknown (*n* = 13 missing responses) (Table 1). Regarding metastatic status at diagnosis, 209 patients (74.9%) reported no metastases, 17 (6.1%) reported metastases, and 53 (19%) were uncertain (*n* = 7 missing responses). No patient reported bone, brain, or skin metastases; however, hepatic metastases were reported by three patients and pulmonary metastases by one patient. Fifteen patients listed “other” metastatic sites. At the time of the survey, 45 patients (15.7%) were receiving ongoing therapy, including chemotherapy (*n* = 10), antihormonal therapy (*n* = 4), PARP inhibitor therapy (*n* = 3), and bevacizumab (*n* = 1) (*n* = 20 missing responses) (Table 1).

### 3.2. Course of Symptoms

Patients were asked about symptoms at the time of initial diagnosis, with multiple responses permitted. The majority of patients were symptomatic (*n* = 178, 62.9%). The most common complaints were pain (*n* = 94, 33.2%), abdominal distension (*n* = 89, 31.4%), and menstrual irregularities (*n* = 43, 15.2%). Approximately a third of the patients (*n* = 105, 36.7%) were asymptomatic at the time of diagnosis. Three patients did not provide a response to this question. A complete list of reported symptoms is presented in Table 2. Among the 31 patients (11.4%) with recurrent disease at the time of data collection, most were asymptomatic at recurrence (*n* = 14/31, 45.2%), followed by pain (*n* = 8/31, 25.8%). Other symptoms reported at recurrence included bowel symptoms (*n* = 3/31, 9.7%), abdominal distension (*n* = 3/31, 9.7%), menstrual irregularities (*n* = 3/31, 9.7%), weight loss (*n* = 3/31, 9.7%), dyspnea (*n* = 2/31, 6.5%), and other symptoms (*n* = 9/31, 29%). Multiple responses were permitted (Table 2). At the time of study enrollment, 93 patients (32.5%) reported experiencing symptoms, while 185 were asymptomatic. Reported complaints included pain (*n* = 57, 20.5%), urinary symptoms (*n* = 14, 5%), bowel symptoms (*n* = 25, 9.0%), abdominal distension (*n* = 18, 6.5%), menstrual irregularities (*n* = 4, 1.4%), weight loss (*n* = 3, 1.1%), dyspnea (*n* = 7, 2.5%), and other symptoms (*n* = 21, 7.6%) (*n* = 8 missing responses) (Table 2).

### 3.3. Risk and Protective Factors

Prior to the initial diagnosis of borderline ovarian tumor, 93 patients (32.8%) had a known ovarian cyst. In 161 patients (56.7%), no cyst had been identified and 30 patients (10.6%) reported the status as unknown (*n* = 2 missing responses). The majority of patients had used hormonal contraception (*n* = 225, 79.2%), most commonly for up to 10 years (*n* = 133, 46.8%). A total of 92 patients (32.4%) reported use for more than 10 years, while 59 patients (20.8%) had not used hormonal contraception prior to the initial diagnosis. Two patients did not provide a response. A total of 69 patients (26.2%) reported a positive family history of breast cancer (no: *n* = 185, 70.3%; unknown: *n* = 9, 3.4%; not answered: *n* = 23), and 20 patients (8.3%) reported a positive family history of ovarian cancer (no: *n* = 213, 88.4%; unknown: *n* = 8, 3.3%; not answered: *n* = 45) (Table 3). Patients were also asked whether they had undergone genetic testing for a BRCA mutation. The majority had not been tested (*n* = 177, 78.7%), while 33 patients (14.7%) reported having undergone testing (Table 3). For 14 patients (6.2%), the testing status was unknown, and 61 patients did not provide a response. Among those who underwent genetic testing (*n* = 33, 100%), 24 patients (72.7%) reported a negative result and 3 patients (9.1%) reported a positive result. For four patients (12.1%), the BRCA test result was unknown, and two patients did not provide a response (Table 3).

### 3.4. Reproductive Outcomes

**Figure 1:**
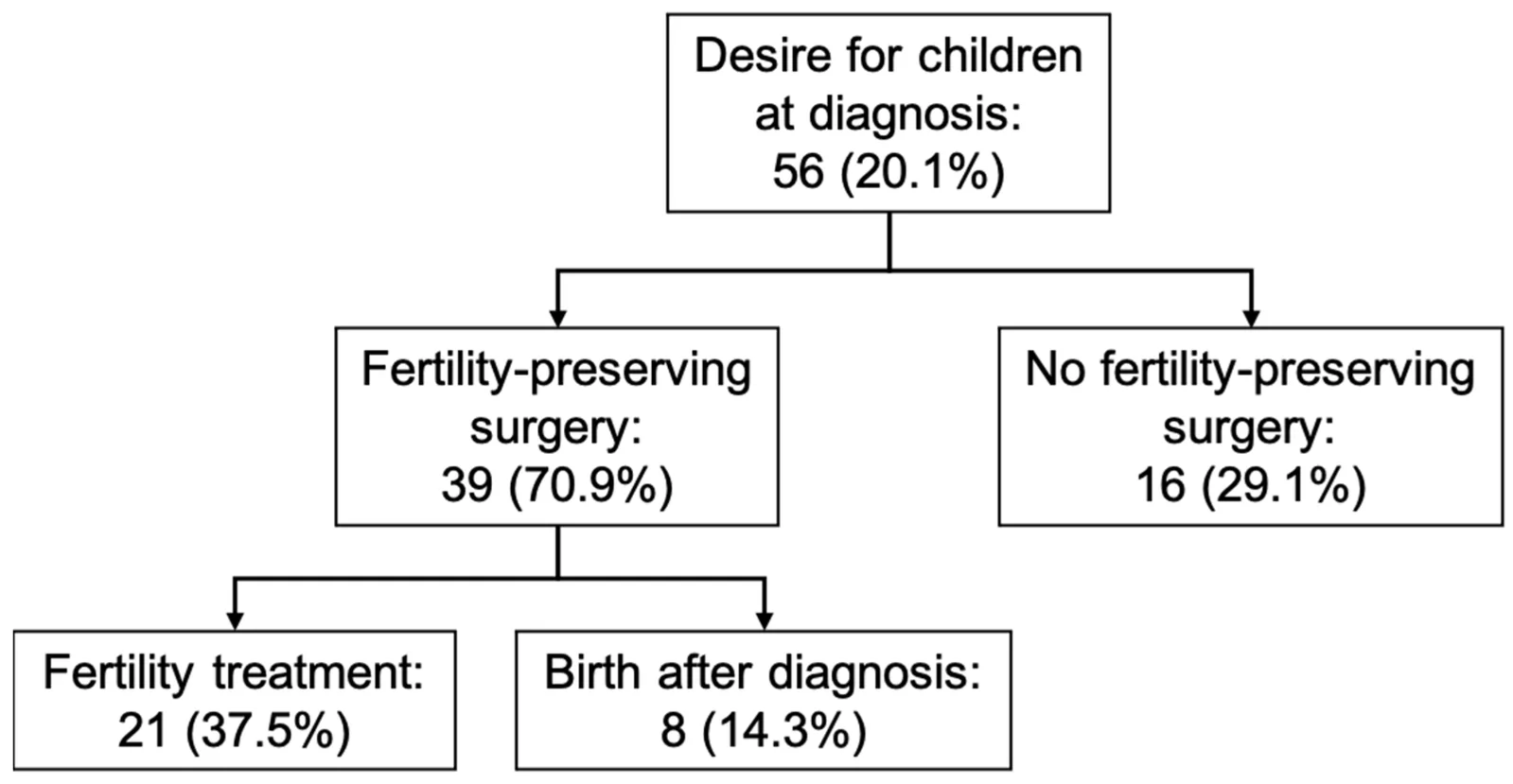
Flowchart of reproductive outcome. Total cohort: 286, percentages refer to respondents; *n* = 278 for this item; 8 missing; missing response regarding surgery: *n* = 1.

Among the 286 patients, the number of pregnancies ranged from 0 to 12. Most patients reported having been pregnant twice (*n* = 83, 29.3%), closely followed by those who had never been pregnant (*n* = 80, 28.3%). Information on the number of births was provided by 283 patients. The mean number of births was 1.27 (SD 1.20), with a median of 1.0 (range 0–7). Three patients did not provide a response. At the time of initial diagnosis, 56 patients (20.1%) expressed a desire to have children (*n* = 8 missing responses). Among this subgroup, fertility-preserving surgery was performed in 39 patients (70.9%), while 16 patients (29.1%) declined such a procedure and one patient did not provide a response. Furthermore, 21 patients (37.5%) in this subgroup had undergone or were undergoing fertility treatment at the time of the study, and 8 patients (14.3%) had given birth following the initial diagnosis (see Figure 1).

### 3.5. Level of Patient Education About Their Disease, Treatment, and Follow-Up Care

Information regarding the source from which patients received most information about their disease, prognosis, and treatment options was collected. The treating physician was by far the most frequently reported source (*n* = 228, 80.9%). Twelve patients (4.3%) cited the internet, four (1.4%) mentioned a nurse, three (1.1%) a family member, and one (0.4%) obtained most information from another patient. Sixteen patients (5.7%) selected “other,” and four did not respond.

Patients rated the quality of information provided by their physicians regarding the disease using a 10-point rating scale. Responses were obtained from 276 of 286 patients (96.5%). The mean score was 8.6 ± 1.7, with a median of 9 (IQR 8–10, range 1–10), and 72% of patients gave scores between 8 and 10 (Table 4). Regarding therapy, the information was rated similarly: 253 patients (88.5%) responded, with a mean score of 8.3 ± 2.1, a median of 9 (IQR 8–10, range 1–10), and 72% selecting scores between 8 and 10 (Table 4).

Regarding knowledge of cancer type, 179 patients (63%) correctly indicated that they did not have ovarian, fallopian tube, or peritoneal cancer, whereas 83 (29%) believed they did and 24 (8%) were uncertain (Table 5). When asked about borderline ovarian tumors—explained as tumors exhibiting cellular characteristics typical of malignancy, but lacking invasive growth—250 patients (87%) answered “Yes,” 26 (9%) “No,” and 10 (4%) “Unknown” (Table 5).

At initial diagnosis, 170 patients (63.9%) were unaware of their tumor stage and 96 (36.1%) reported knowing it (*n* = 20 missing responses) (Table 6). In total, 69 patients (25.9%) were able to report their FIGO stage: stage I (*n* = 39/69, 56.5%), stage II (*n* = 10/69, 14.5%), stage III (*n* = 18/69, 26.1%), and stage IV (*n* = 2/69, 2.9%) (Table 6). Information on pT stage was available for 26 patients (9.8%; pT1: *n* = 7/26, 26.9%; pT2: *n* = 7/26, 26.9%; pT3: *n* = 12/26, 46.2%; pT4: *n* = 0). Only 38 patients (14.3%) provided information on both FIGO and pT stage (Table 6).

**Figure 2:**
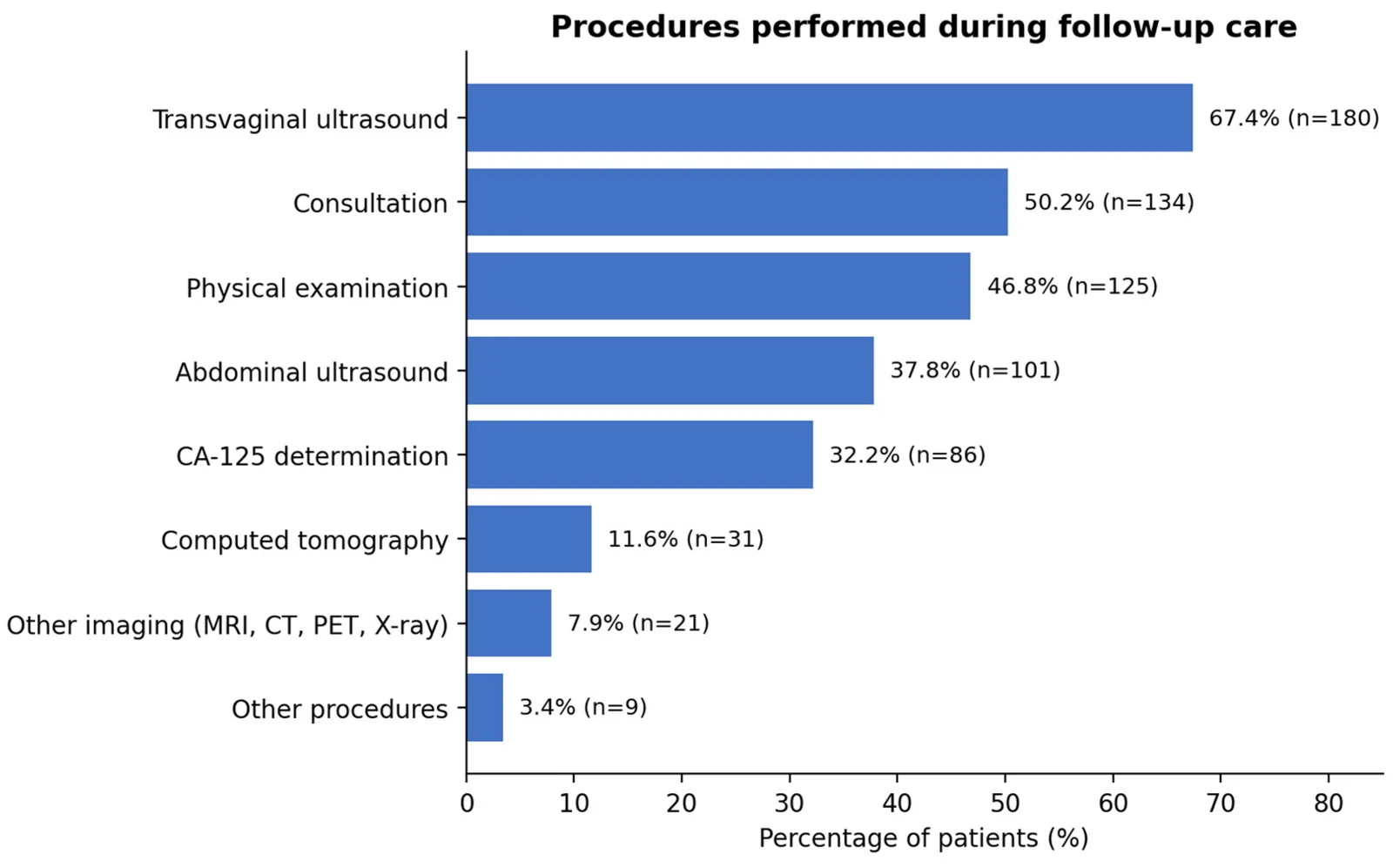
Procedures performed during follow-up care. Note: Percentages may not sum to 100%, as multiple procedures could be reported per patient.

Concerning follow-up care, 277 patients (96.9%) reported receiving regular follow-up, while 9 (3.1%) did not. Procedures performed during follow-up included consultation (*n* = 134, 50.2%), physical examination (*n* = 125, 46.8%), transvaginal ultrasound (*n* = 180, 67.4%), abdominal ultrasound (*n* = 101, 37.8%), computed tomography (*n* = 31, 11.6%), other imaging modalities (e.g., MRI, CT, PET, X-ray; *n* = 21, 7.9%), determination of CA-125 (*n* = 86, 32.2%), and other procedures (*n* = 9, 3.4%), as shown in Figure 2. A total of 47 patients (17.6%) were unaware of which examinations were performed, and 19 did not respond.

### 3.6. Illness Perception

Patients were asked which terminology describing their disease elicited the greatest anxiety. The majority indicated that “ovarian tumor of low malignant potential” was perceived as equally distressing as “borderline ovarian tumor” (*n* = 103, 37.7%). In sum, 88 patients (32.3%) reported that neither term caused them anxiety, 41 (15%) considered “ovarian tumor of low malignant potential” most distressing, and another 41 (15%) regarded “borderline ovarian tumor” as most distressing. Thirteen patients did not respond.

**Figure 3:**
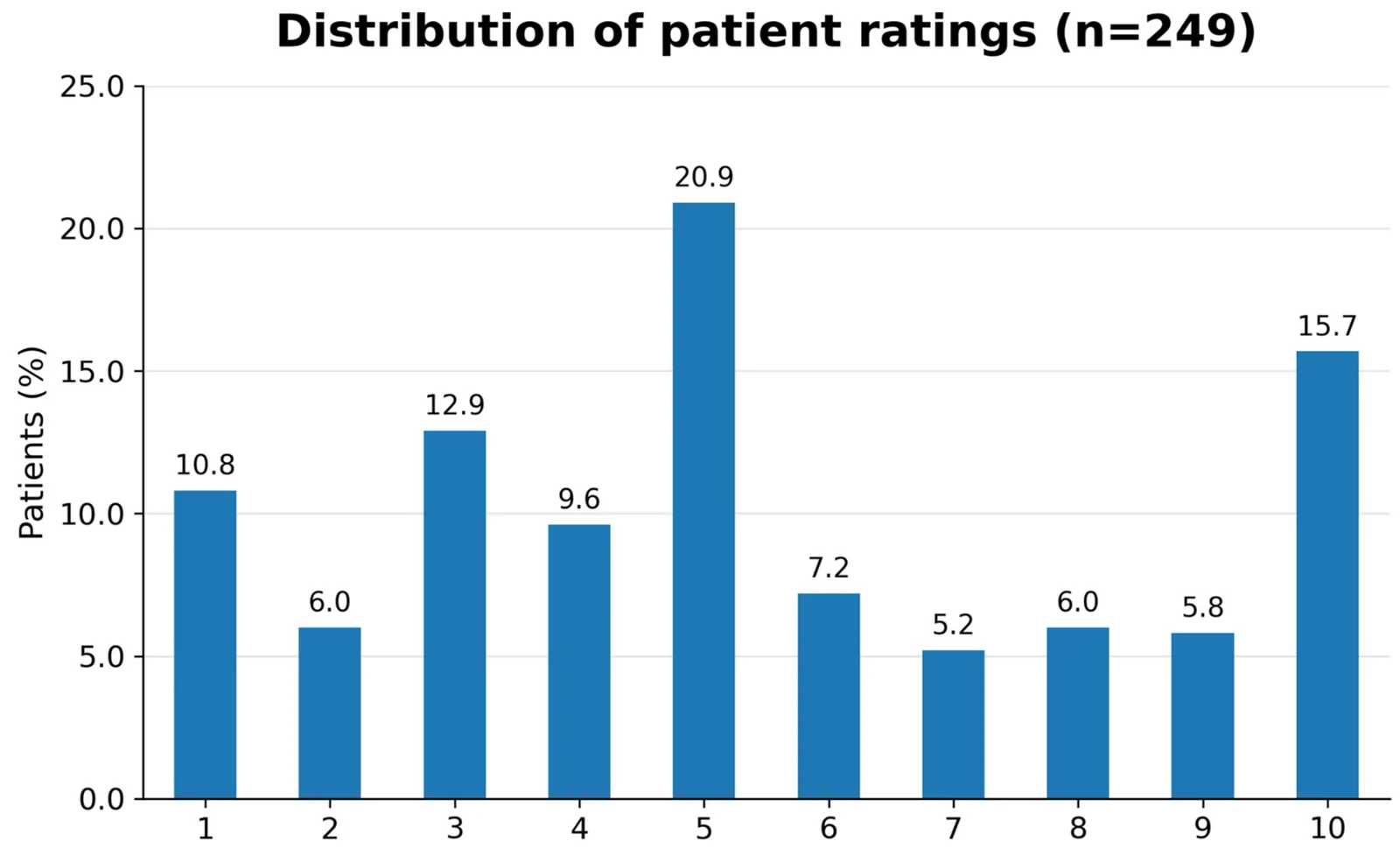
Distribution of patient ratings of perceived disease severity on a 1–10 scale (*n* = 249). Patients were asked to rate their condition from 1, representing a benign cyst, to 10, representing ovarian cancer.

**Figure 4:**
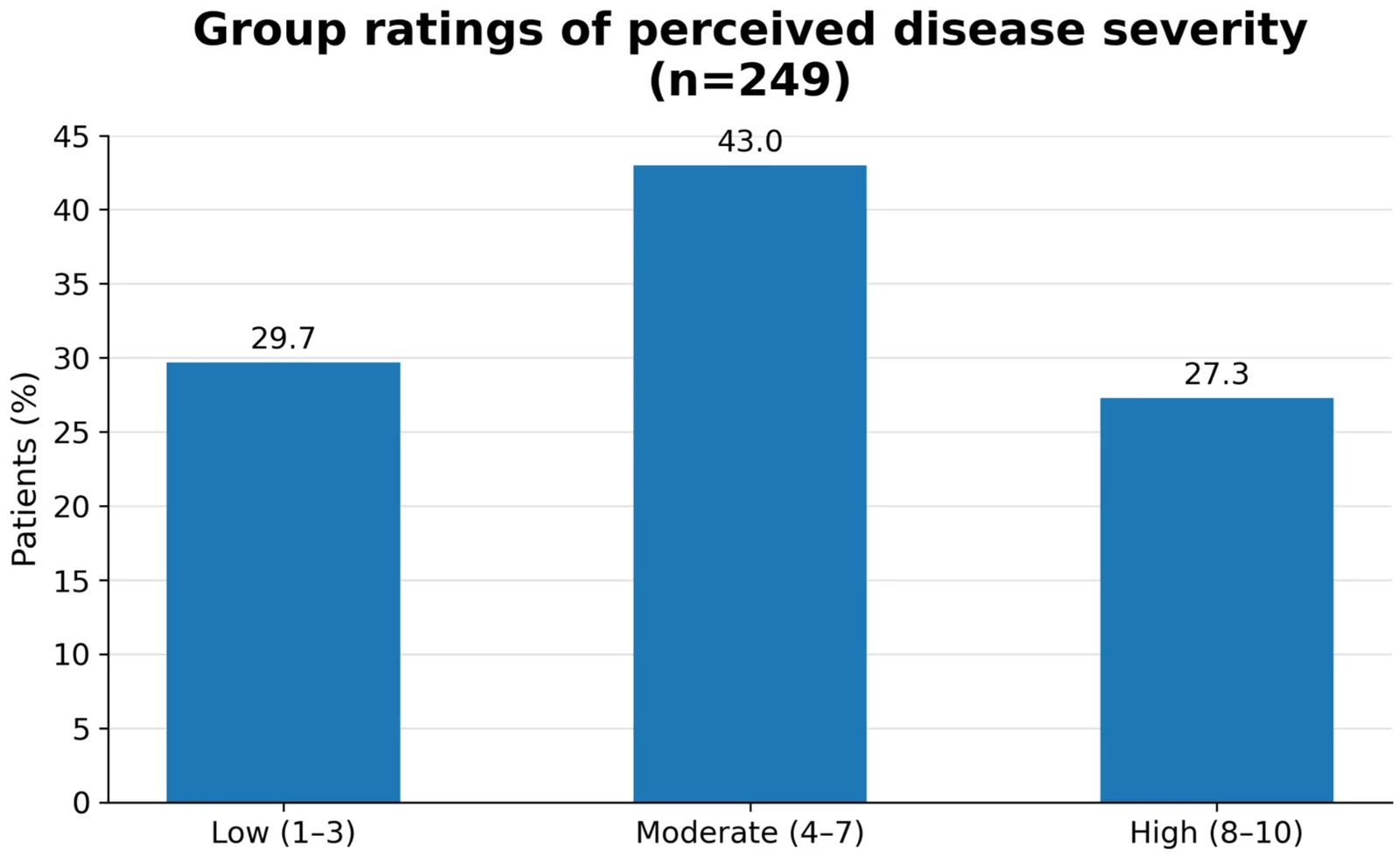
Grouped ratings of perceived disease severity (*n* = 249). Low severity was defined as scores 1–3, moderate severity as scores 4–7, and high severity as scores 8–10.

Perceived disease severity was assessed on a scale from 1 to 10, where 1 indicated a benign cyst and 10 indicated ovarian cancer. Of the 286 patients, 249 (87.1%) completed this scale. The mean rating was 5.4 ± 2.9, with a median of 5 (IQR 3–8; range 1–10) (Figure 3). Nearly a third of patients (29.7%) rated their condition as low severity (scores 1–3), 43.0% as moderate (scores 4–7), and 27.3% as high (scores 8–10) (Figure 4).

**Figure 5:**
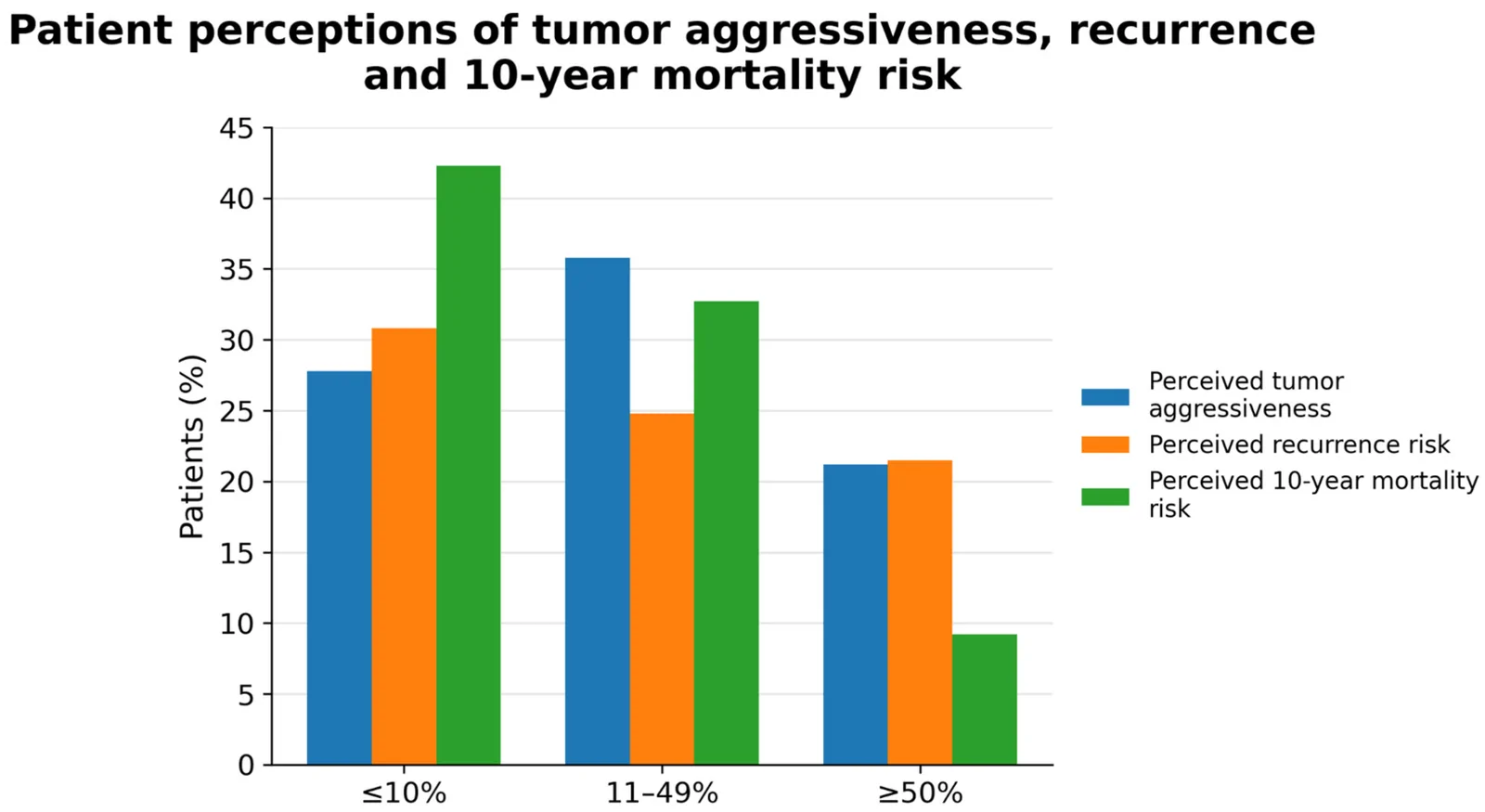
Patient perceptions evaluated on a 0–100% scale of tumor aggressiveness, recurrence, and 10-year mortality risk. For better interpretability, responses were categorized into three ranges: ≤10% (perceived as rather benign), 11–49% (intermediate perception), and ≥50% (perceived as rather malignant).

Patients also estimated tumor aggressiveness as a percentage from 0 to 100%, without further specification of what aggressiveness entails, allowing patients to apply their own understanding of the term. Of 286 patients, 245 (85.7%) provided a response. The mean perceived aggressiveness was 31.7 ± 27.4%, with a median of 20% (IQR 10–50%; range 0–100%). Nearly a third of patients rated aggressiveness at ≤10%, whereas approximately a quarter estimated it at ≥50% (Figure 5).

Perceived risk of tumor recurrence was evaluated on a 0–100% scale. Of 286 patients, 247 (86.4%) responded. The mean perceived risk of recurrence was 28.6 ± 30.0%, with a median of 20% (IQR 2–50%; range 0–100%). More than a third (36.4%) estimated a risk of 10% or lower, while approximately a quarter (25.5%) reported estimates of 50% or higher (Figure 5).

Finally, patients were asked to estimate the 10-year risk of dying from the disease. Responses were obtained from 243 of 286 patients (85.0%). The mean perceived 10-year mortality risk was 18% (SD ~23%), with a median of 5% (IQR 0–20%; range 0–100%). Notably, half of the patients (50.2%) indicated a risk of 0%, whereas about 11% reported estimates of 50% or higher (Figure 5).

## 4. Discussion

This study aimed to assess how patients with borderline ovarian tumors are informed about their disease, how they perceive and understand it, and to provide additional insights into the clinical course, including fertility intentions and symptom burden. To our knowledge, this represents the largest cohort to date investigating patient awareness and disease perception in this specific population. A strength of this study is that it assessed patient-reported outcomes in a large multicenter cohort of patients with borderline ovarian tumors, a rare disease entity for which data on patients’ perspectives are still limited. The questionnaire allowed a comprehensive evaluation of disease understanding, perception, information needs, and physician–patient communication, none of which can be captured through clinical data alone. The standardized and anonymous survey design across 34 centers may also have made it easier for patients to openly report their individual experiences and concerns.

Consistent with previous reports [19,20,21,22,23], our study confirms that the treating physician remains the primary source of information for patients, with 80.9% citing their physician as their main informational resource, while—contrary to expectations—only 4.3% considered the internet relevant despite the young age of the cohort [24]. Nonetheless, substantial knowledge gaps were observed: 29% incorrectly believed they had cancer and 63.9% were unaware of their tumor stage at diagnosis. Although 72% of patients rated the quality of physician-provided information between 8 and 10, these persistent misconceptions may partly reflect uncertainties of the treating physicians themselves, which are inadvertently conveyed to patients. As a limitation, these findings reflect self-reported data and are therefore subject to recall bias. This is particularly relevant given that a substantial proportion of patients were unaware of their exact disease stage.

Fotopoulou et al. examined patients’ perceptions of their disease in a cohort of 60 women in 2010, demonstrating that patients perceived the malignant potential of borderline ovarian tumors to be comparable to that of invasive ovarian cancer, with an equally high perceived risk of recurrence [14]. These perceptions appear to have changed little over time. In our larger cohort, which included a more comprehensive assessment of disease perception, 70.3% of patients rated disease severity as moderate to high, approximately a quarter overestimated tumor aggressiveness, 25.5% estimated a recurrence risk greater than 50%, and 11% anticipated a 50% or higher probability of dying from the disease within 10 years. These findings underscore the ongoing need to improve patient education on the biological behavior and pathogenesis of borderline ovarian tumors, enabling a more accurate understanding of their disease and its management.

Although borderline ovarian tumors are typically diagnosed at an early stage, often incidentally during routine examinations, nearly two-thirds of patients in our cohort (62.9%) reported symptoms at diagnosis. The most frequent complaints were abdominal pain (33.2%) and abdominal distension (31.4%), consistent with previous reports that patients often present with non-specific abdominal symptoms [25,26]. Given that 32.8% had a previously known ovarian cyst, the coexistence of cystic lesions and abdominal symptoms should prompt close clinical follow-up to enable early detection and timely surgical intervention.

Surgery remains the cornerstone of treatment, reflected by 94% of patients undergoing surgical management at diagnosis. Optimal staging includes peritoneal cytology, resection of suspicious lesions, peritoneal biopsies, and omentectomy, irrespective of whether a radical or fertility-sparing approach is pursued [12,27,28]. As incomplete staging is associated with higher recurrence risk [7,11], the finding that only 44.4% reported omentectomy and 86.1% ovarian removal suggests either suboptimal staging or limited patient awareness of surgical details. A limitation of the questionnaire is that it did not allow respondents to specify whether one or both ovaries were removed, nor to indicate if a tumor resection alone, with preservation of the ovary, had been performed. Under optimal surgical management, a higher proportion of patients would have been expected to undergo both ovary removal and omentectomy, highlighting the potential gaps in either treatment or patient awareness. Lymphadenectomy was performed in 14.3% of patients, indicating potential overtreatment contrary to current recommendations [28,29,30]. Overall, 15.4% of patients reported receiving adjuvant systemic therapy—most commonly chemotherapy (9%) or bevacizumab (4.2%)—despite the lack of indication for such treatment in this entity [27,28,31,32]. These findings reflect ongoing uncertainty among treating physicians regarding surgical extent and adjuvant management, emphasizing the need for centralization of care in specialized centers to ensure adherence to current guidelines and optimize outcomes.

Borderline ovarian tumors affect predominantly younger women compared to invasive ovarian carcinomas, and are most often diagnosed at an early disease stage [3,4,5]. Consequently, fertility preservation is a central aspect of management. Current ESGO–ESMO and national (AWMF) guidelines recommend fertility-sparing surgery for unilateral disease and may also be considered acceptable in patients with advanced-stage disease, particularly in younger patients desiring future childbearing [8,9,27,28]. However, as fertility-sparing surgery has been identified as an independent risk factor for recurrence, it should be offered after thorough counseling and shared decision-making with the patient [8,33,34,35]. Regarding the age distribution of our patient cohort, 27% of patients were between 18 and 41 years old. At the time of initial diagnosis, 20.1% of patients reported a desire for future childbearing. However, fertility-sparing surgery was performed in only 39 patients—corresponding to 14% of the total cohort and just 70.9% of patients who expressed a desire for children. Given the explicit recommendations of current national and international guidelines, the observed rates are notably lower than expected.

Evidence guiding follow-up in ovarian disease remains limited; however, clear recommendations exist regarding the procedures that should be performed during surveillance [27,36]. Interestingly, 96.9% of patients reported receiving regular follow-up; however, only 50.2% considered physician consultation to be part of their follow-up, and 17.6% stated that they were unaware of what was performed during their follow-up visits. These findings again highlight substantial gaps in patient education, which should be addressed in routine clinical practice and in future studies.

## 5. Conclusions

Taken together, these findings provide important insights into patient perceptions, knowledge, and communication patterns in the context of borderline ovarian tumors, and they highlight key opportunities for improving patient education and clinical management. Although overall communication with physicians was rated positively, persistent misconceptions among BOT patients underscore the need for clearer, structured education on the biological behavior and evidence-based management of the disease, including the distinction between borderline tumors and carcinoma. Strengthening adherence to clinical guidelines and further centralizing care in specialized centers may help minimize overtreatment and optimize fertility-preserving outcomes. However, centralization alone is unlikely to close the communication and knowledge gaps identified in this study. This would additionally require a more structured approach to ensuring that patients have genuinely understood the information conveyed to them, which is also a precondition for meaningful shared decision-making.

## Figures and Tables

**Table 1:** Patient Characteristics.

**No. of patients**	286
**Age group, *n* (%)**	
18–40 years	76 (27.0%)
41–50 years	41 (14.5%)
51–60 years	71 (25.2%)
61–70 years	61 (21.6%)
≥71 years	33 (11.7%)
**Height (cm), mean ± SD**	166.1 (±6.7)
**Weight (kg)**	75.2 (±19.0)
**BMI (kg/m^2^)**	27.3 (±6.7)
**Stage of disease**	
Primary disease	210 (76.9%)
Relapsed disease	31 (11.4%)
Unknown	32 (11.7%)
**Treatment at initial diagnosis**	
Surgery	266 (94.0%)
Chemotherapy	25 (9%)
Maintenance therapy with bevacizumab	11 (4.2%)
Antihormonal therapy	6 (2.2%)
Current treatment	
Yes	45 (16.9%)
No	210 (79%)
Unknown	11 (4.1%)

**Table 2:** Symptomatology at initial diagnosis, recurrence, and study enrollment. Patients reported symptoms at three time points. Multiple responses were allowed. Percentages are based on the number of respondents to each question (*n* = 283 at initial diagnosis, *n* = 31 at recurrence, and *n* = 278 at the time of data collection).

Symptom	Initial (*n*, %)	Recurrence (*n*, %)	Study Enrollment (*n*, %)
Pain	94, 33.2%	8, 25.8%	57, 20.5%
Abdominal distension	89, 31.4%	3, 9.7%	18, 6.5%
Bowel symptoms	-	3, 9.7%	25, 9.0%
Urinary symptoms	-	-	14, 5.0%
Menstrual irregularities	43, 15.2%	3, 9.7%	4, 1.4%
Weight loss	-	3, 9.7%	3, 1.1%
Dyspnea	-	2, 6.5%	7, 2.5%
Other	-	9, 29%	21, 7.6%
Asymptomatic	105, 37.1%	14, 45.2%	185, 66.6%

**Table 3:** Patients’ family history regarding breast and ovarian cancer and potentially genetic testing performed. Percentages are calculated based on valid responses, excluding missing responses.

Characteristic	*n*	%
**Family history of breast cancer**		
Yes	69	26.2%
No	185	70.3%
Unknown	9	3.4%
Not answered	23	—
**Family history of ovarian cancer**		
Yes	20	8.3%
No	213	88.4%
Unknown	8	3.3%
Not answered	45	—
**Genetic testing for BRCA mutation**		
Yes	33	14.7%
No	177	78.7%
Unknown	14	6.2%
Not answered	61	—
**BRCA test result (among tested patients, *n* = 33)**		
Negative	24	72.7%
Positive	3	9.1%
Unknown	4	12.1%
Missing	2	—

**Table 4:** Patient-rated quality of information provided by physicians (10-point rating scale, 1 = very poor, 10 = very good).

Topic	Responses, *n* (%)	Mean ± SD	% Scoring 8–10
Disease information	276 (96.5%)	8.6 ± 1.7	72%
Therapy information	253 (88.5%)	8.3 ± 2.1	72%

**Table 5:** Patient knowledge of cancer status and understanding of borderline ovarian tumor. * Borderline ovarian tumor was explained as a tumor exhibiting cellular characteristics typical of malignancy, but lacking invasive growth.

Response	*n*	%
**Belief regarding having cancer**		
Correctly identified as not having cancer	179	63%
Believed they had cancer	83	29%
Uncertain	24	8%
Understanding of borderline ovarian tumor definition *		
Yes	250	87%
No	26	9%
Unknown	10	4%

**Table 6:** Patient-reported knowledge of tumor stage at initial diagnosis. Note: Only 38 patients (14.3%) provided information on both FIGO and pT stage.

	*n*	%
**Awareness of tumor stage (*n* = 266; 20 missing)**		
Unaware of stage	170	63.9%
Aware of stage	96	36.1%
**FIGO stage reported (*n* = 69/267, 25.9%)**		
Stage I	39/69	56.5%
Stage II	10/69	14.5%
Stage III	18/69	26.1%
Stage IV	2/69	2.9%
**pT stage reported (*n* = 26/267, 9.8%)**		
pT1	7/26	26.9%
pT2	7/26	26.9%
pT3	12/26	46.2%
pT4	0/26	0%

## Data Availability

The data presented in this study are available from the corresponding author upon reasonable request. Due to ethical and privacy restrictions, patient-level data cannot be publicly shared.

## Abbreviations

The following abbreviations are used in this manuscript.

BOT	borderline ovarian tumor
NOGGO	North-Eastern German Society of Gynecologic Oncology
SD	standard deviation
IQR	interquartile range
